# Supplementary material for: Cascadable all-optical NAND gates using diffractive networks
Source: Sci Rep. 2022 May 3;12:7121. doi: 10.1038/s41598-022-11331-4 (PMC9065113; doi:10.1038/s41598-022-11331-4)
Supplement: Supplementary file 1 — Supplementary Information. [file 41598_2022_11331_MOESM1_ESM.pdf]

Cascadable all-optical NAND gates using diffractive networks

Yi Luo[1,2,3] e-mail: yluo2016@ucla.edu

Deniz Mengu[1,2,3] e-mail: denizmengu@g.ucla.edu

Aydogan Ozcan[1,2,3*] e-mail: ozcan@ucla.edu

[1]Electrical and Computer Engineering Department, University of California, Los Angeles, California 90095, USA

[2]Bioengineering Department, University of California, Los Angeles, California 90095, USA

[3]California Nano Systems Institute (CNSI), University of California, Los Angeles, California 90095, USA

[*]Correspondence: Prof. Aydogan Ozcan

E-mail: ozcan@ucla.edu

Address: 420 Westwood Plaza, Engr. IV 68-119, UCLA, Los Angeles, CA 90095, USA

Tel: +1(310)825-0915

Fax: +1(310)206-4685

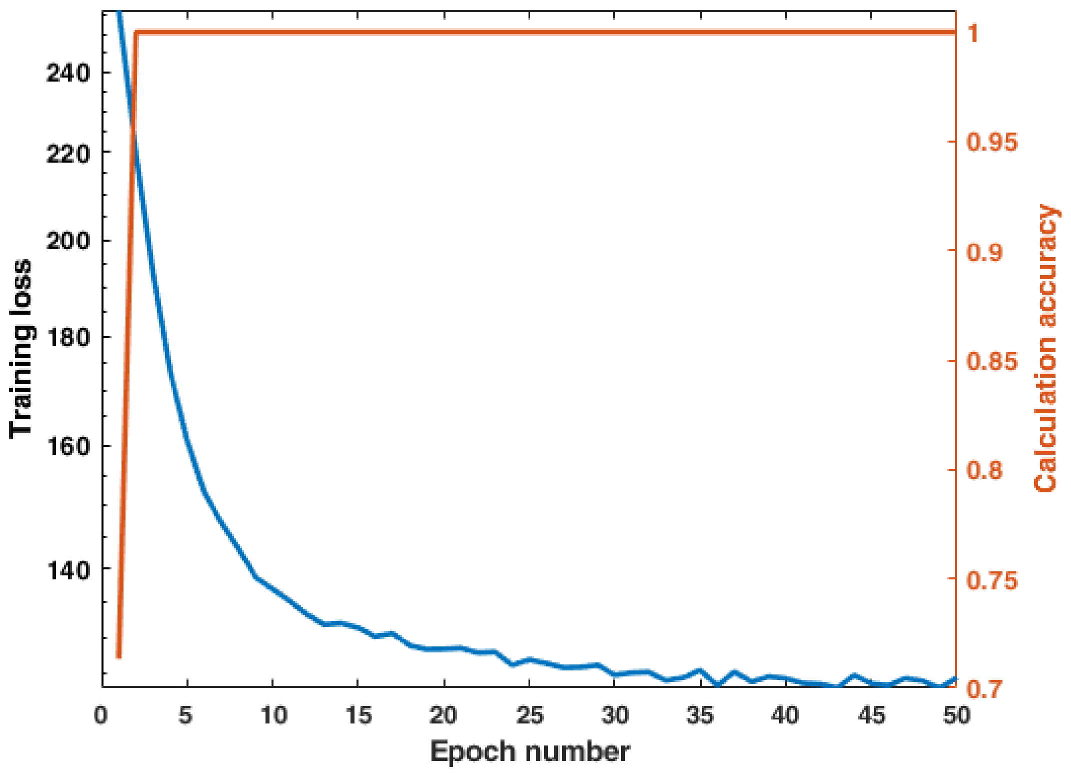

Supplementary Figure S1. Training log of the diffractive NAND gate.
